# A sensory-motor neuron type mediates proprioceptive coordination of steering in *C*. *elegans* via two TRPC channels

**DOI:** 10.1371/journal.pbio.2004929

**Published:** 2018-06-08

**Authors:** Jihye Yeon, Jinmahn Kim, Do-Young Kim, Hyunmin Kim, Jungha Kim, Eun Jo Du, KyeongJin Kang, Hyun-Ho Lim, Daewon Moon, Kyuhyung Kim

**Affiliations:** 1 Department of Brain and Cognitive Sciences, DGIST, Daegu, Republic of Korea; 2 Companion Diagnostic Medical Technology Research Group, DGIST, Daegu, Republic of Korea; 3 Department of Structure & Function of Neural network, KBRI (Korea Brain Research Institute), Daegu, Republic of Korea; 4 Department of Anatomy and Cell Biology, Sungkyunkwan University School of Medicine, Suwon, Republic of Korea; 5 Department of New Biology, DGIST, Daegu, Republic of Korea; Rockefeller University, United States of America

## Abstract

Animal locomotion is mediated by a sensory system referred to as proprioception. Defects in the proprioceptive coordination of locomotion result in uncontrolled and inefficient movements. However, the molecular mechanisms underlying proprioception are not fully understood. Here, we identify two transient receptor potential cation (TRPC) channels, *trp-1* and *trp-2*, as necessary and sufficient for proprioceptive responses in *C*. *elegans* head steering locomotion. Both channels are expressed in the SMDD neurons, which are required and sufficient for head bending, and mediate coordinated head steering by sensing mechanical stretches due to the contraction of head muscle and orchestrating dorsal head muscle contractions. Moreover, the SMDD neurons play dual roles to sense muscle stretch as well as to control muscle contractions. These results demonstrate that distinct locomotion patterns require dynamic and homeostatic modulation of feedback signals between neurons and muscles.

## Author summary

Proprioception provides the nervous system with feedback about body posture in animals and is essential for the generation of coherent locomotive behaviors, such as walking, running, or crawling. However, little is known about the identity of proprioceptive receptors that sense body movement to regulate locomotion and the extent to which proprioception modulates sensorimotor coordination. Here, we analyze the molecular mechanisms that control head steering locomotion of *Caenorhabditis elegans*. We show that this movement is regulated by the transient receptor potential cation (TRPC) channels TRP-1 and TRP-2 and the SMDD proprioceptive neurons. We observe that mutant animals for both channels are defective in head steering locomotion and that ectopic expression of TRP-1 or TPR-2 in a *C*. *elegans* chemosensory neuron confers head bending–dependent responses, suggesting roles for these channels in proprioception. We also find that SMDD neurons are both necessary and sufficient to generate head steering locomotion via the two channels. Moreover, we demonstrate that the proprioceptive system mediates locomotion coordination by desynchronizing activities in motor systems. We conclude that two TRPC channels in collaboration with the proprioceptive receptor SMDD neurons control head steering in worms during forward locomotion.

## Introduction

Animal locomotive behaviors, which include crawling, walking, swimming, and running, are mediated by a sensory system referred to as proprioception [1–3]. Specialized proprioceptive neurons sense body and limb movements and send signals to the brain, in which the proprioceptive signals are integrated and processed to coordinate motor activity [2–4]. In invertebrates, proprioceptive signals are detected by proprioceptive neurons and proprioceptors, which are located within the body wall muscles or mechanosensitive organs and orchestrate the movement of each body segment [5–9]. Similarly, in mammals, proprioceptors are located in their muscles, ligaments, and joints to coordinate the movements of the limbs [3, 10]. Defects in the proprioception-mediated coordination of locomotion cause uncontrolled and inefficient movements such as ataxic gait [7, 8, 11], but the precise molecular mechanisms by which proprioception occurs and how it modulates sensorimotor coordination remain unclear. Specifically, the cells and stretch-sensitive molecules that mediate proprioception need to be fully identified.

Mechanosensitive channels, including transient receptor potential (TRP) channels, degenerin/epithelial sodium channels (DEG/ENaC), and Piezo have been implicated as putative proprioceptors that transduce the mechanical signals derived from muscle and limb movements into electrical signals [8, 12–15]. TRP channels have been well characterized in animal models and in vitro expression systems as cellular sensors that detect a variety of mechanical forces. For example, *Drosophila* transient receptor potential no mechanical potential C (TRPN/NompC) channels are expressed in the bipolar dendrite (bd) and class I dendritic arborization (da) proprioceptive neurons of chordotonal organs, in which they are involved in both larval crawling and adult locomotion [8, 12, 16]. In addition, heterologously expressed transient receptor potential ankyrin (TRPA), transient receptor potential mucolipin (TRPM), transient receptor potential cation (TRPC), and transient receptor potential vanilloid (TRPV) channel isoforms can be activated by membrane stretch [17–24], but their precise functional role in proprioception remains unclear.

The nematode *C*. *elegans* is a genetically tractable animal model in which to dissect the sensorimotor feedback system’s underlying locomotion. Its complete synaptic wiring of motor circuits [25, 26] and a broad spectrum of locomotive behaviors, including forward and backward crawling and swimming [27, 28], provide a unique opportunity to study molecular and neuronal mechanisms underlying locomotive behaviors at single-synapse resolution. *C*. *elegans* moves in a sinusoidal wave pattern via a periodic bending of its head and body. These movements are thought to be generated and shaped by proprioception [29]. Two putative types of proprioceptive neurons have been identified: the B-type cholinergic neurons, which may respond to local body bending and propagate proprioceptive signals along the body, and the DVA neurons, which regulate the extent of body bending via activation of *trp-4* TRPN channels [13, 30]. However, the molecular nature of the stretch-sensitive proprioceptive receptors and the mechanism by which these neurons direct proprioceptive feedback systems have not yet been identified.

Here, we show that the *C*. *elegans* TRPC channels *trp-1* and *trp-2* are both necessary for the proprioception-mediated steering of forward locomotion and sufficient for stretch-induced neuronal responses upon expression. *trp-1 trp-2* double-mutant animals show defects in steering during forward movement, leading to ventral circling locomotion. Both TRP-1 and TRP-2 are expressed in the SMDD proprioceptive neurons, the ablation of which also causes ventral circling locomotion. The activity of the SMDD neurons induced by head bending is not abolished but instead misregulated in *trp-1 trp-2* mutants, and optogenetic manipulation of the activity of SMD neurons similar to that observed in *trp-1 trp-2* double mutants also results in ventral circling locomotion. Moreover, expression of the proprioceptive receptors *C*. *elegans trp-4* or *Drosophila trpγ* rescues the locomotive defects of *trp-1 trp-2* double mutants, and ectopic expression of TRP-1 or TPR-2 causes robust Ca^2+^ responses to head bending in a *C*. *elegans* chemosensory neuron. Together, these results reveal that sensorimotor coordination of head steering locomotion in *C*. *elegans* is mediated by two TRPC channels *trp-1* and *trp-2* in the SMDD proprioceptive neurons.

## Results

### *trp-1 trp-2* double mutants make ventral-directed circles during forward movement

**Fig 1:**
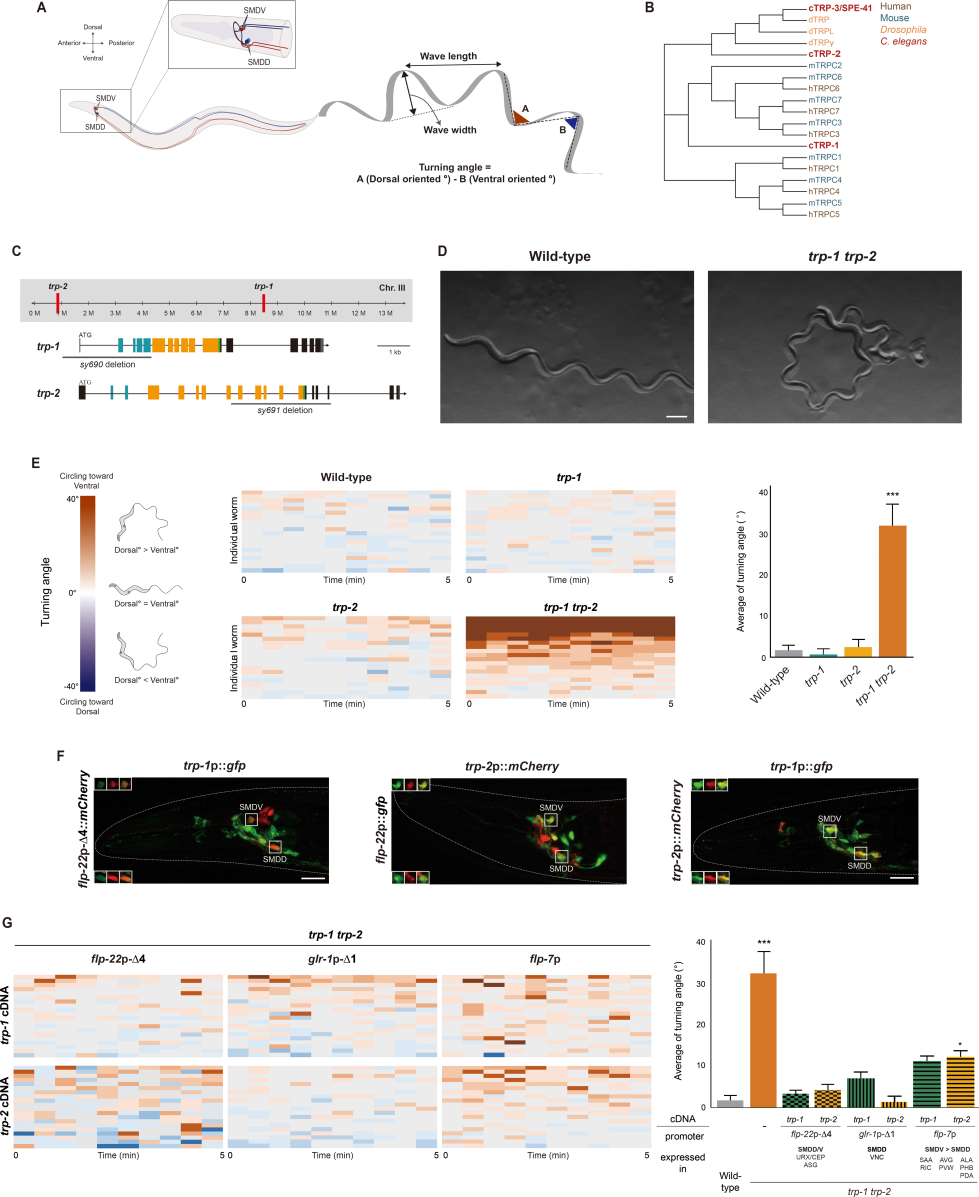
TRP-1 and TRP-2 are required for head steering locomotion. (**A**) Schematic of the SMD neurons in *C*. *elegans* and the values analyzed during forward movement. **(B)** A phylogenetic tree of the TRPC subfamily channels. **(C)** The genomic locations (above) and structures (below) of the *trp-1* and *trp-2* genes. The gray lines indicate the regions deleted in the *trp-1 (sy690)* and *trp-2 (sy691)* mutants. Ankyrin repeats, transmembrane domains, and TRP domains are labeled in blue, yellow, and green, respectively. **(D)** Sinusoidal waveform tracks of wild-type animals (left) and *trp-1 trp-2* double-mutant animals (right) on a bacterial lawn. Scale bar: 25 μm. **(E)** Heat maps and a color-coded average turning angle for the indicated genotypes. *n* = 20 for each genotype. **(F)** Expression patterns of the indicated transgenes. Images in the upper left and lower left boxed regions are single-focal-plane confocal microscopy images of the SMDV and SMDD soma, respectively. Anterior is to the left. Scale bar: 25 μm. **(G)** Heat maps and the average turning angle for the indicated genotypes. *n* = 20 for each. Numerical values that underlie the graph are shown in S1 Data. Error bars indicate SEM. * and *** indicate a significant difference from wild type at *p* < 0.05 and *p* < 0.001, respectively (one-way ANOVA test followed by the Tukey post hoc test). TRP, transient receptor potential; TRPC, transient receptor protein cation; VNC, ventral nerve cord

To identify the factors that mediate proprioception to modulate the sinusoidal waveforms of *C*. *elegans*, we performed a candidate mutant screen of mechanosensitive TRP channels and DEG/ENaC genes [31–33]. We measured 44 mutant strains, including 19 TRP channels and 25 DEG/ENaC channel genes, for three values of the sinusoidal locomotive waveforms including wave width, wave length, and turning angle (Fig 1A, S1–S3 Figs). We found several mutant strains with altered values (S1–S3 Figs). Since the defects were mild, however, we decided not to pursue these mutants further.

Instead, we began to generate double mutants for genes examined in the first screening. The *C*. *elegans* genome contains three TRPC channel genes (i.e., *trp-1*, *trp-2*, and *trp-3/spe-41*; Fig 1B), of which *trp-3* is expressed exclusively in sperm [32–34]. To determine whether remaining TRPC family members regulate locomotion, we used genetic recombination to create *trp-1 trp-2* double mutants. In the *trp-1 (sy690)* allele, the promoter and the majority of the N-terminus of the TRPC channel are missing; in the *trp-2 (sy691)* allele, half the transmembrane domain is lost along with the TRP box domain. The nature of these molecular lesions suggests that they are likely null alleles (Fig 1C). We found that *trp-1 trp-2* (*sy690 sy691*) double mutants exhibited continuous circular sinusoidal locomotion (Fig 1D, S1 and S2 Videos). To quantitate this circling behavior, we measured their turning angle and found it to be significantly higher than that of wild-type animals (*trp-1 trp-2*: 31.9° ± 5.1°, *n* = 20; wild type: 1.71° ± 1.1°, *n* = 20; Fig 1E). We call this phenotype “ventral circling locomotion.” In contrast to the double mutants, *trp-1* or *trp-2* single mutants did not exhibit ventral circling locomotion (Fig 1E). While the wave width of the *trp-1 trp-2* double mutants is similar to that of wild-type animals, their wave length is slightly reduced (S4 Fig). The ventral circling locomotion of *trp-1 trp-2* double mutants persists in the absence of bacterial food (S5 Fig). However, we did not find any differences of turning angles between wild-type and *trp-1 trp-2* mutant animals during reversals (S6 Fig). Together, these results suggest that *trp-1* and *trp-2* regulate the turning angle of forward movement.

### *trp-1* and *trp-2* are expressed and act in the SMD neurons to modulate the curvature of forward movement

Next, we generated transgenic animals expressing *trp-1*p::*gfp* or *trp-2*p::*mCherry* transgenes under the control of 2.6-kb and 3-kb promoter regions, respectively [35–37]. We found that *trp-1* and *trp-2* were coexpressed in several head neurons, including the putative proprioceptive SMD neurons (Fig 1F) [26]. The SMD neurons include two left and right pairs of neurons (i.e., the dorsal SMDDL/R and ventral SMDVL/R) with cell bodies in the head that extend processes sublaterally from head to tail to innervate the muscles of the head. The SMDV cell bodies are located dorsally, and they send processes subventrally, whereas the SMDD cell bodies are located in the ventral ganglion, and their processes follow other dorsal sublateral cords (Fig 1A) [26]. To verify the expression of *trp-1* and *trp-2* in the SMD neurons, we compared the expression of the *trp-1* and *trp-2* reporters to that of the *flp-22*p*-Δ*4 and *flp-22* SMD reporters (Fig 1F). The *flp-22*p*-Δ*4 promoter resides upstream of the *flp-22* promoter, which drives transgene expression in several head neurons, including ASG and CEP (or URX), as well as in SMD (S7A and S7B Fig) [38]. In this study, we used the *flp-22*p*-Δ*4 promoter to express transgenes in the SMD neurons, except when otherwise noted. We observed strong overlap between the *trp-1* or *trp-2* transgene and SMD reporter expression (Fig 1F). We also found colocalization of *trp-1* expression with *trp-2* in the SMD neurons (Fig 1F), indicating that both *trp-1* and *trp-2* are indeed expressed in the SMD neurons.

To determine whether the ventral circling locomotion is caused by loss of *trp-1* and *trp-2* function in SMD, we expressed the *trp-1* cDNA or *trp-2* cDNA under the control of the *flp-22*p*-Δ*4 promoter in the *trp-1 trp-2* double-mutant background. We found that expression of either *trp-1* cDNA or *trp-2* cDNA rescued the locomotion defects of the *trp-1 trp-2* double mutants (Fig 1G, S8A Fig). We further dissected their relative contributions of the dorsal and ventral SMD neurons by expressing the *trp-1* cDNA or *trp-2* cDNA specifically in the SMDD and/or SMDV neurons under the control of the *glr-1*p*-Δ*1 and *flp-7* promoters, respectively. The *glr-1*p*-Δ*1 promoter drives transgene expression in SMDD and some ventral nerve cord neurons but not in SMDV neurons; the *flp-7* promoter drives expression consistently in several neurons, including SMDV but also weakly and occasionally in SMDD (S8B Fig) [38, 39]. Interestingly, *glr-1*p*-Δ*1 promoter–driven expression of either TRP-1 or TRP-2 fully rescued the ventral circling phenotype of the *trp-1 trp-2* double mutants, but *flp-7* promoter–driven expression produced a weaker rescue (Fig 1G, S8A Fig). These results suggest that TRP-1 and TRP-2 act in SMDD to regulate turning angle of forward locomotion.

### TRP-1 and TRP-2 synchronize SMDD neuronal activity with dorsal head/neck bending

**Fig 2:**
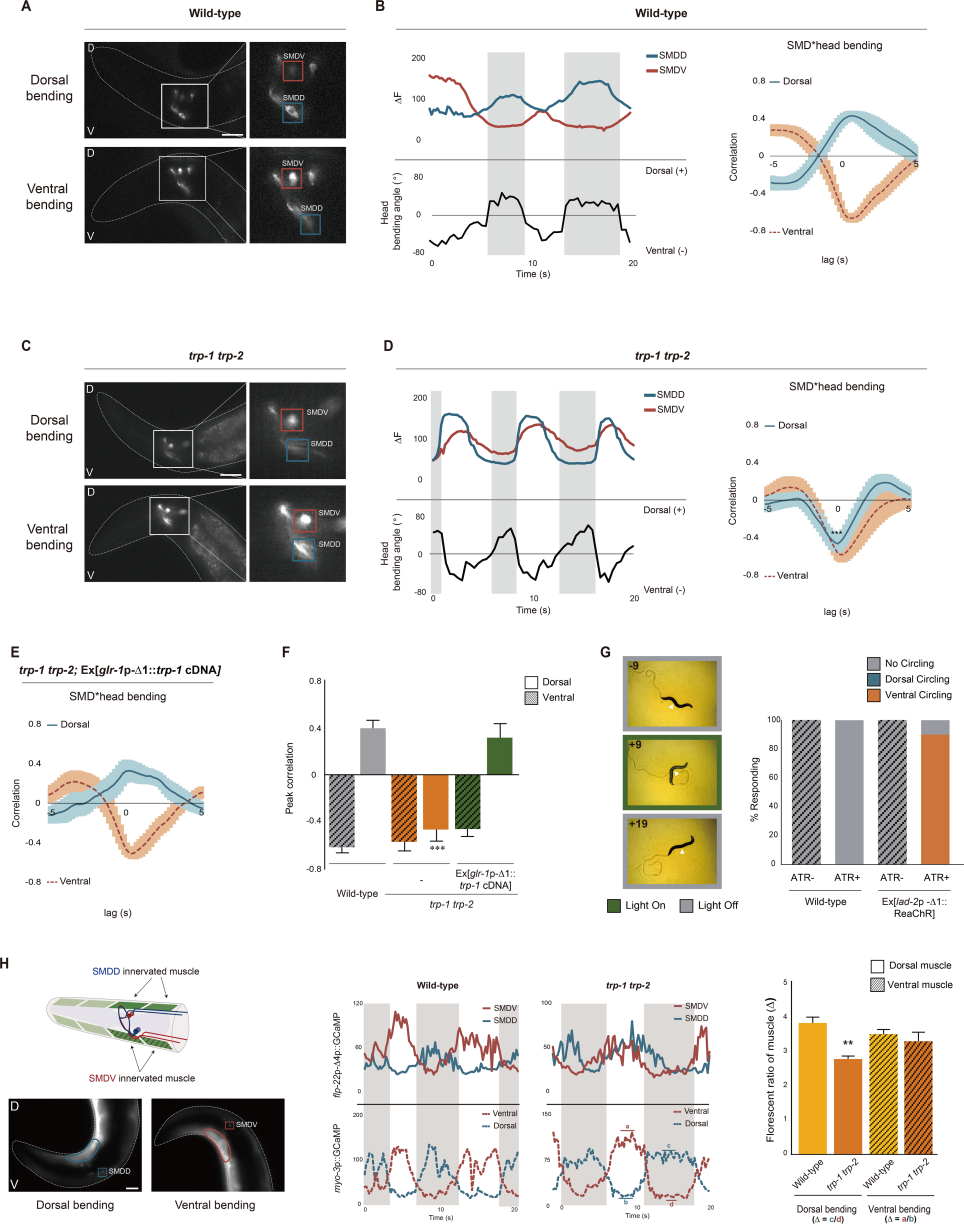
TRP-1 and TPR-2 coordinate SMD neuronal activities with head bending and mediate head muscle contractions. **(A, C)** Representative images showing GCaMP3 fluorescence in the SMD soma of a *flp-22*p*-Δ*4::GCaMP3 transgenic worm during dorsal and ventral head bending of the indicated genotypes. The head region (left) and a higher magnification of the boxed area (right) are shown. Red and blue boxes indicate the cell bodies of SMDV and SMDD, respectively. Anterior is to the left. Scale bar: 25 μm. **(B, D, E)** Calcium dynamics (top left) in the SMD cell bodies and the corresponding head bending (bottom left) in the same animal and cross-correlations (right) between SMDD or SMDV calcium responses and head bending **(B, D)** and cross-correlations of the indicated genotype **(E)**. Gray bars indicate the duration of the dorsal head bending. *n* = 10 for each. **(F)** Peak correlations between SMD calcium activity and head bending of the indicated genotypes. Peak correlation value is obtained from lag 0 of cross-correlation. **(G)** Representative images showing a *lad-2*p-*Δ*1::ReaChR::mKate2 transgenic animal upon green light stimulation (left) and the percentage of animals that exhibit circling locomotion in response to light stimulation in the presence and absence of retinal (ATR; right). Arrowheads indicate the ventral side of the body. *n* = 40. **(H)** Schematic of the head muscles innervated by SMDD and SMDV (left), single frame images of head muscles of *myo-3*p::GCaMP3.35; *flp-22*p-*Δ*4::GCaMP3 transgenic worms during head bending (middle), and the GCaMP fluorescent ratio of dorsal muscles to ventral muscles (right). D: dorsal; V: ventral. Anterior is to the left. Scale bar: 25 μm. *n* = 10 for each genotype. Numerical values that underlie the graph are shown in S1 Data. Error bars indicate SEM. ** and *** indicate significant differences from wild type at *p* < 0.01 and *p* < 0.001, respectively (one-way ANOVA test followed by the Tukey post hoc test). ATR, all-trans-retinal; TRP, transient receptor potential

To investigate the physiological roles of TRP-1/TRP-2 in the SMD neurons during forward locomotion, we first generated transgenic animals expressing GCaMP3 in the SMD neurons and recorded the Ca^2+^ transients of all the SMDD and SMDV somas as the animals moved freely. We observed oscillating Ca^2+^ transients in SMD during movement; the SMDD calcium activity was increased during dorsal head bending and reduced during ventral head bending, whereas the SMDV calcium transients were increased during ventral head bending and reduced during dorsal head bending (Fig 2A and 2B, S9 Fig, S3 Video) [40, 41]. These results indicate that the SMDD and SMDV oscillating Ca^2+^ waves are in antiphase (Fig 2B). A cross-correlation analysis of the SMD calcium dynamics and head bending showed a strong correlation between SMDD calcium activity and dorsal head bending and between SMDV activity and ventral head bending (Fig 2B and 2F).

Next, we found that the SMDV and SMDD neurons of *trp-1 trp-2* double mutants also exhibited oscillating Ca^2+^ transients during forward movement. However, although we did not see any changes in the calcium activity of the SMDV neurons, the Ca^2+^ transients of SMDD in *trp-1 trp-2* double mutants were reverse-correlated with dorsal head bending and positively correlated to ventral head bending. This leads to two oscillating Ca^2+^ waves in SMDV and SMDD with phases similar to each other (Fig 2C, 2D and 2F, S9 Fig, S4 Video). We also noted that SMDD activity is a little lower than SMDV activity in *trp-1 trp-2* double mutants (S10A Fig). The *glr-1*p*-Δ*1 promoter–driven expression of the *trp-1* cDNA fully rescued this defect in the SMDD calcium dynamics (Fig 2E and 2F). The SMD activities of the *trp-1* or *trp-2* single mutants showed strong correlations with head bending like wild-type animals (S10B Fig), consistent with no apparent phenotype in forward locomotion upon each single mutation. These results suggest that TRP-1 and TRP-2 play a role in coordinating the activity of the SMDD neurons with dorsal head movement.

Since the oscillatory calcium transients of SMDD and SMDV are synchronized in the *trp-1 trp-2* mutants, we next asked whether the simultaneous activation of SMDD and SMDV can induce a locomotion pattern that mimics the defect of the *trp-1 trp-2* mutants. We performed optogenetic experiments using transgenic animals that express the channelrhodopsin variant ReaChR::mKate2 in the SMDD/V neurons under the control of the *lad-2*p-*Δ*1 promoter [42, 43]. The *lad-2*p-*Δ*1 promoter is expressed in ALN, PLN, SAA, SDQ, and SMDD/V (S7C Fig). Previous studies have shown that optogenetic activations of the PLN, ALN, and SDQ neurons do not affect forward movement [44], nor do ablations of the SAA neurons [37]. Upon green light stimulation in the presence of retinal, transgenic worms instantly began ventral bending, which led to a ventral circling phenotype like that of the *trp-1 trp-2* mutants (Fig 2G, S5 Video). Upon termination of the light stimulation, the animals immediately reverted to normal forward movement. These results suggest that the ventral circling phenotype of the *trp-1 trp-2* double mutants is due to the synchronization of the activities of SMDV and SMDD neurons.

SMDD and SMDV innervate the dorsal and ventral head muscles near the nerve ring region (Fig 2H) [26]. Previously, it was shown that a set of cholinergic marker genes are expressed in the SMD neurons and that a few excitatory nicotinic acetylcholine receptors are expressed in muscle [45, 46], suggesting that synaptic transmission from the SMD neurons to muscle is excitatory [47, 48]. To verify that synaptic transmission between SMD and neck muscles is excitatory, we performed the calcium imaging and optogenetic experiments by expressing ReaChR channelrhodopsin in the SMD neurons and measuring Ca^2+^ transients in dorsal and ventral muscles upon light exposure. Upon light exposure, Ca^2+^ transients in both dorsal and ventral muscles near the neck were strongly increased (S11 Fig), indicating that synaptic transmission from the SMD neurons to neck muscles is indeed excitatory. Then, we speculated that the uncoordinated SMDD calcium dynamics with head movement in the *trp-1 trp-2* mutants impair dorsal head muscle contractions, leading to the ventral circling locomotion phenotype. Using transgenic animals expressing GCaMP3.35 in body wall muscles [49], we measured GCaMP intensity in the head muscles while observing SMD Ca^2+^ dynamics with head bending (Fig 2H, S12 Fig). We found that Ca^2+^ levels in the dorsal head muscles of *trp-1 trp-2* double mutants during dorsal bending, when the SMDD GCaMP fluorescence is brightest, were reduced compared to those of wild-type animals, whereas the ventral muscle levels are unaltered (Fig 2H, S12 Fig). In addition, we examined the general morphology of the body wall muscles of wild-type, *trp-1 trp-2* double-mutant, and *unc-89* mutant animals, using label-free coherent anti-Stokes Raman scattering microscopy [50]. *unc-89* gene encodes a component of the M-line and organizes the myosin filament structure of muscles in *C*. *elegans* [51]. Compared to disorganized muscle structure of *unc-89* mutants, wild-type and *trp-1 trp-2* mutant animals exhibit linearized and well-organized sarcomere structure (S13 Fig), indicating that the reduced Ca^2+^ activity in the dorsal head muscles is not a result of structural defects in the muscles. Consistent to this observation, *trp-1* and *trp-2* genes are not expressed in muscles (Fig 1F). Together, these results suggest that TRP-1 and TRP-2 in SMDD are involved in linking head bending to head muscle contractions.

### TRP-1 and TRP-2 may function as stretch-mediated proprioceptors in the SMDD neurons

**Fig 3:**
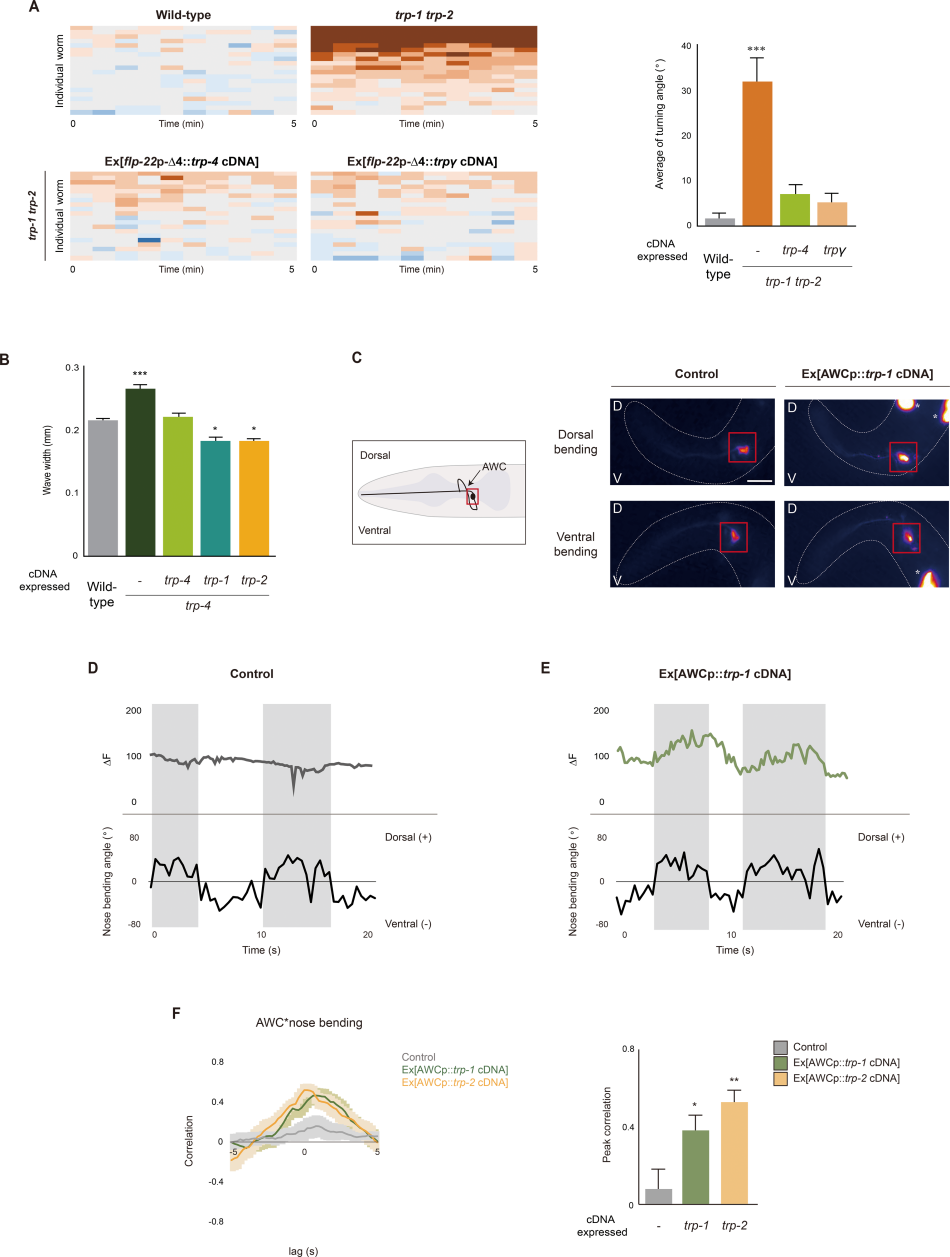
TRP-1 and TRP-2 are stretch-sensitive proprioceptive receptors. **(A)** Heat maps and the average turning angle for indicated genotypes. *n* = 20 for each genotype. **(B)** Average wave width of the indicated genotypes. *n* = 50 for each genotype. **(C)** Schematic of the AWC neuron in the head region (left) and single-frame images of GCaMP3 signals in the AWC soma of control animals and transgenic animals expressing *trp-1* cDNA in AWC during nose bending (right). Red boxes indicate the AWC cell bodies. * indicates fluorescence in the coelomocytes expressing an injection marker. D: dorsal; V: ventral. Scale bar: 25 μm. Anterior is to the left. **(D, E)** Calcium dynamics in AWC cell bodies and the corresponding nose bending of control (D) and transgenic animals expressing *trp-1* cDNA in AWC (E). Gray bar represents the duration of the dorsal head bending. **(F)** Cross-correlations and peak correlations between AWC calcium responses and nose bending of the indicated genotypes. Peak correlation value is obtained from lag 0 of cross-correlation. *n* = 10 for each. Numerical values that underlie the graph are shown in S1 Data. Error bars indicate SEM. *, **, and *** indicate significant differences from wild type at *p* < 0.05, *p* < 0.01, and *p* < 0.001, respectively (one-way ANOVA test followed by the Tukey post hoc test). TRP, transient receptor potential

We next asked whether TRP-1 and TRP-2 function as stretch-sensitive receptors to detect head bending–driven mechanical stretches due to the contraction of head muscles. First, we performed rescue experiments by expression of the previously identified stretch receptors *C*. *elegans* TRP-4 and *Drosophila* TRPγ [8, 13]. *trp-4* encodes a TRPN subfamily member that is expressed in the DVA putative proprioceptive neuron and that regulates body bending [13]; *trpγ* is a TRPC homolog gene expressed in femoral chordotonal proprioceptive organs that regulate fine leg coordination during walking [8]. The expression of the *trp-4* or *trpγ* cDNAs in the SMD neurons fully rescued the ventral circling locomotion defect of *trp-1 trp-2* mutants (Fig 3A). In addition, we expressed the *trp-1* or *trp-2* cDNAs in the DVA neuron of *trp-4 (sy695)* mutants. *trp-4* mutants exhibit exaggerated wave width during forward movement due to defects in body stretch–mediated proprioception [13]. As with the expression of TRP-4, the expression of TRP-1 or TRP-2 in the DVA neuron rescued the altered locomotion of *trp-4* mutants. In addition, the wave width for these animals was even smaller than that of wild-type animals (Fig 3B). These results indicate that the stretch sensitive TRP-4 and TRPγ channels can functionally substitute for TRP-1 and TPR-2. Furthermore, the role of the TRPC channels is evolutionarily conserved between *C*. *elegans* and *Drosophila* [8].

To further investigate the stretch-sensitive functions of TRP-1 and TRP-2, we first ectopically expressed the *trp-1* or *trp-2* cDNAs in the AWC chemosensory neurons of transgenic animals also expressing GCaMP3. We then monitored Ca^2+^ transients in the AWC somas during freely moving conditions. Previous studies have shown that the AWC neurons elicit Ca^2+^ transients upon odor removal [52]. In addition, ultrasound-induced mechanical deformation also induces Ca^2+^ responses in the AWC neurons expressing TRP-4 [53]. We detected weak and inconsistent Ca^2+^ responses in the AWC neurons of forward-moving animals (Fig 3C and 3D) [53]. However, misexpression of either TRP-1 or TRP-2 in the AWC neurons caused the AWC neurons to additionally produce strong and consistent Ca^2+^ responses, depending upon dorsoventral nose bending (Fig 3C and 3E). We observed increases of the AWC Ca^2+^ signal during dorsal nose bending that fell back to baseline during ventral bending, resulting in a strong correlation between the Ca^2+^ transients in the AWC neurons and dorsal nose bending (Fig 3F). Next, we tested two other chemosensory neurons, AWA and ASI, and found that, similar to those in TRP-1-expressed AWC, Ca^2+^ transients in TRP-1-expressed ASI were increased only upon dorsal nose bending but not ventral nose bending (S14 Fig). However, we did not see significant difference of Ca^2+^ transients in TRP-1-expressed AWA (S14 Fig). These results suggest that ectopic expression of TRP-1 or TRP-2 is sufficient to confer neuronal responses upon mechanical deformation of head muscles in a context-dependent manner and support a role for TRP-1 and TRP-2 as putative stretch-sensitive receptors.

### The SMDDs are stretch-sensitive proprioceptive neurons that directly regulate muscle contractions

**Fig 4:**
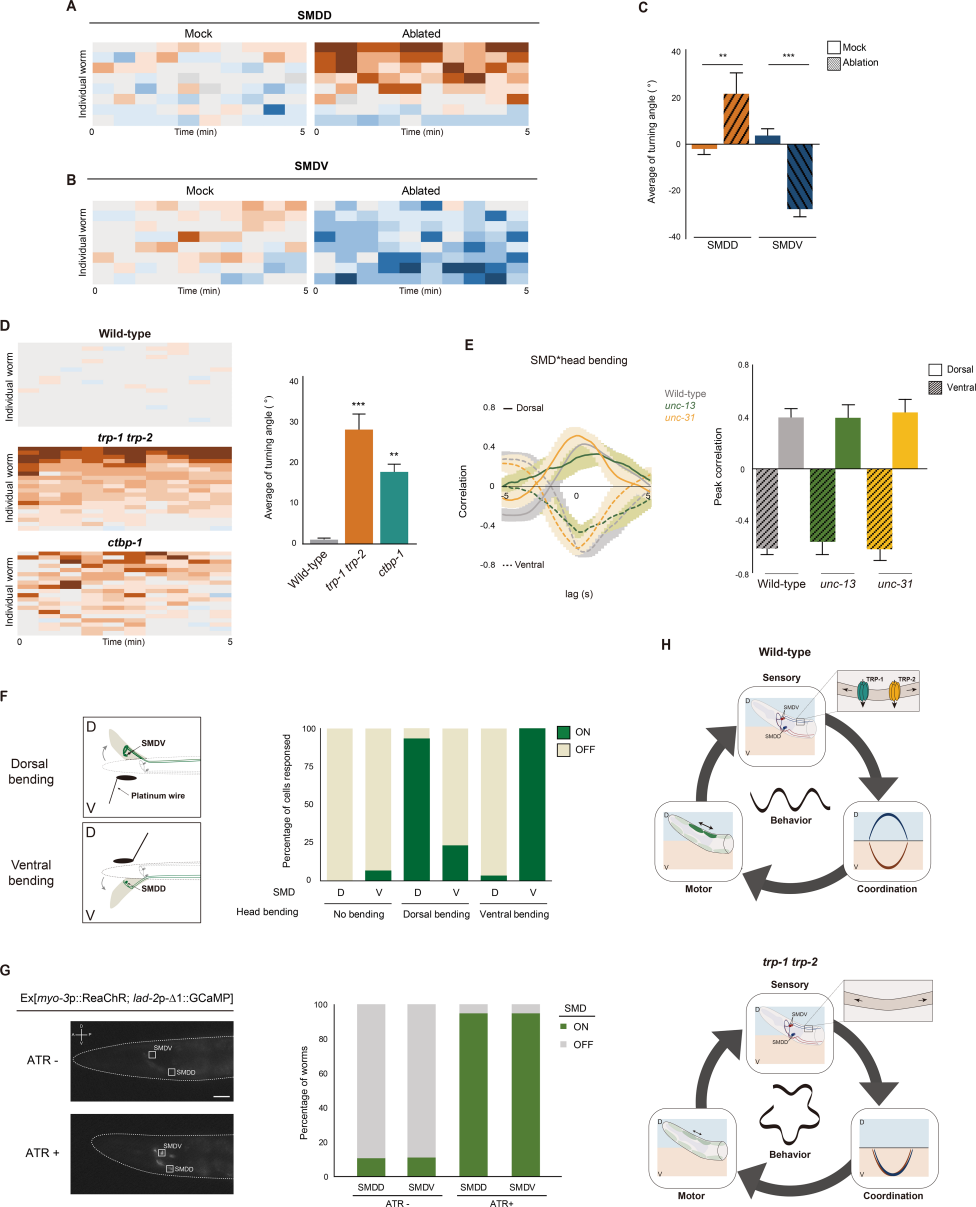
The SMD neurons are stretch-sensitive proprioceptive neurons. **(A-D)** Heat maps (A, B, D) and the average turning angle (C) for the SMDD- or SMDV-ablated animals or indicated genotypes. *n* = 8 for each. **(E)** Cross-correlations and peak correlations of the indicated genotypes. Peak correlation value is obtained from lag 0 of cross-correlation. *n* = 10 for each. **(F)** Schematic for inducing head bending using a platinum wire (left) and the percentage of worms with increased GCaMP3 intensity in SMD soma of *unc-54 (e1092)* mutant animals induced by head bending (right). D: dorsal; V: ventral. *n* = 30 for each. **(G)** Representative images of Ex[*myo-3*p::ReaChR; *lad-2*p-*Δ*1::GCaMP] transgenic animals upon green light stimulation in the presence and absence of retinal (ATR; left) and the percentage of animals that exhibit GCaMP3 signals in the SMD neurons (right). White rectangles indicate soma of SMDD/V. *n* = 40. **(H)** Model for the functions of *trp-1* and *trp-2* in the SMD neurons coordinating neuronal activity with the motor system to regulate turning angle during forward movement. Numerical values that underlie the graph are shown in S1 Data. Error bars indicate SEM. ** and *** indicate significant differences from wild type at *p* < 0.01 and *p* < 0.001, respectively (one-way ANOVA test followed by the Tukey post hoc test). ATR, all-trans-retinal

To determine whether the SMD neurons are stretch-sensitive proprioceptive neurons that mediate forward locomotion, we first performed laser microsurgery to individually ablate pairs of SMDD or SMDV cell bodies. Ablation of the SMDD cell bodies resulted in a ventral circling phenotype similar to that observed with the *trp-1 trp-2* mutants, confirming that the ventral circling phenotype of the *trp-1 trp-2* double mutants is due to a functional defect in the SMDD neurons (Fig 4A and 4C). Furthermore, ablation of the SMDV neurons induces dorsal circling locomotion (Fig 4B and 4C). Because *ctbp-1* mutants show defects in the neuronal processes of SMDD but not SMDV neurons [39], we examined the locomotion of *ctbp-1* mutants. C-terminal binding protein 1 (CTBP-1) is a transcriptional repressor of C-terminal binding protein, which regulates neuronal development [39]. We found that *ctbp-1* (*ok498*) mutant animals exhibited the ventral circling phenotype like the *trp-1 trp-2* double mutants (Fig 4D). These results indicate that the SMD neurons play a role in steering forward locomotion—SMDD for the dorsal direction and SMDV for the ventral direction.

Because SMD neurons have pre- and postsynaptic connections with several sensory neurons, motor neurons, and interneurons in the head region [26], we asked whether Ca^2+^ transients in the SMD neurons are elicited by transmissions from presynaptic neurons or by direct sensing of head muscle contractions. We first measured Ca^2+^ transients in the SMD neurons of *unc-13 (e1091)* and *unc-31 (e928)*, which have defects in synaptic vesicle release and dense core vesicle release, respectively [54, 55]. We found that although these mutant animals have uncoordinated movements, the correlation between head bending and the activity of their SMD neurons was quantitatively unaltered compared to wild type (Fig 4E). This suggests that SMD Ca^2+^ signals could be induced by direct mechanical sensory stimuli or electric synaptic transmission rather than by chemical synaptic transmission from other cells. We next asked whether the head muscle contractions upon head bending indeed generate SMD calcium activity. When we tested *unc-54 (e1092)* myosin heavy chain mutants, which are paralyzed because of severe defects in muscle contractions [56], we found a complete absence of SMD calcium transients (Fig 4F). We did observe SMDD and SMDV calcium activity in these animals, however, when we physically induced head bending by gently pushing the head with a platinum wire in the dorsal and ventral directions, respectively (Fig 4F). To validate that head muscle contractions are sufficient to generate SMD calcium dynamics further, we generated transgenic animals expressing ReaChR channelrhodopsin in the body wall muscle and calcium sensor GCaMP in the SMD neurons, respectively. The worms were placed into the 4% agar pad to be immobilized. Then, we activated both dorsal and ventral body wall muscles simultaneously by exposing light to animals and observed the SMD Ca^2+^ transients. We found that upon light exposure, Ca^2+^ level was increased in the SMDD and SMDV neurons (Fig 4G), suggesting muscle contractions are indeed sufficient to activate the SMD neurons. Taken together, these results indicate that head muscle contractions are sufficient to directly generate SMD calcium dynamics.

## Discussion

The motor circuits and neural mechanisms underlying the sinusoidal forward and backward movements of *C*. *elegans* have been extensively studied [57, 58]. Little is known, however, about how worms steer their head to maintain a straight path overall. Here, we have determined that the proprioceptive receptor SMD neurons and TRP-1/TRP-2 TRPC channels are required for the proprioceptive feedback that regulates head steering during forward movement.

The SMD cholinergic neurons were suggested to be proprioceptive receptor neurons because of their morphology, including their extension of synapse-free processes along the body and their innervation of head muscles [26, 47]. With several lines of evidence, we demonstrated that the SMD neurons do indeed function as proprioceptive neurons that control head steering locomotion. First, laser ablations of the SMD neurons cause severe defects in steering during forward movement. Killing either the SMDD or the SMDV neurons results in ventral or dorsal circling locomotion, respectively. In addition, the *ctbp-1* mutants, which have defects in their SMDD processes, also exhibit ventral circling locomotion. Second, optogenetic activation of the SMD neurons results in head steering locomotion. When we stimulate all four SMD neurons together, producing a synchronized calcium dynamic similar to that observed in *trp-1 trp-2* double mutants, the animals exhibit ventral circling movements. Third, we found that SMD neurons are activated by head/neck muscle stretching. Forced head/neck bending in the dorsal or ventral directions elicits robust Ca^2+^ transients in SMDD or SMDV neurons, respectively. Finally, disruption of chemical synaptic transmission does not affect the oscillating Ca^2+^ transients produced in the SMD neurons upon head bending. Together, these results indicate the SMD neurons are necessary and sufficient to generate head steering locomotion and strongly support that the SMD neurons are bona fide proprioceptive neurons in *C*. *elegans*.

The DVA neuron, which was previously identified as a proprioceptive neuron, is stimulated by body bending and expresses a stretch receptor, the TRP-4 TRPN channel [13]. Rather than innervating any muscles, however, the DVA neurons appear to send sensory signals to the ventral nerve cord motor neurons to modulate the locomotor circuitry [13, 26]. Additionally, laser ablation of the DVA neurons only mildly affects body bending [13]. Unlike the DVA neurons, the SMD neurons directly sense head/neck muscle stretch and regulate muscle contractions, suggesting head steering locomotion in *C*. *elegans* is regulated mainly by a feedback system utilizing only a single neuronal cell type. In addition, recent studies have shown that SMD regulates the activity of other interneurons, including postsynaptic RIAs and extrasynaptic RMEs via cholinergic neurotransmission to set the amplitude of head bending [40, 41]. This indicates dual roles for SMD in head locomotion: the regulation of steering via direct muscle contractions and the modulation of head bending amplitude via synaptic transmission to other postsynaptic target neurons.

Previously, the TRP-1 and TPR-2 TRPC channels were shown to modulate nicotine-dependent behaviors by acting in command interneurons as receptor-operated channels [36]. Here, we identified a novel role for the TRP-1 and TRP-2 channels as putative stretch-sensing molecules in the proprioceptive SMDD neurons that regulate the proprioceptive feedback system that directs forward locomotion in *C*. *elegans*. First, *trp-1* and *trp-2* are coexpressed in the SMDD proprioceptive receptor neurons. Second, *trp-1 trp-2* double mutants, but not single mutants, exhibit a ventral circling phenotype similar to that of SMDD-ablated animals. This indicates that TRP-1 and TRP-2 are redundant but necessary for SMDD-mediated head steering locomotion. Third, either TRP-1 or TRP-2 is sufficient to confer head/neck bending–dependent Ca^2+^ signals in the AWC chemosensory neuron. Fourth, TRP-1 and TRP-2 are functionally interchangeable with the two known proprioceptors *C*. *elegans trp-4* or *Drosophila trpγ*. Although we could not completely rule out the possibility of an indirect and modulatory role of TRP-1/TRP-2 in stretch sensation [59–61], we suggest that TRP-1 and TRP-2 are putative proprioceptor channels that act redundantly to detect head/neck bending in the SMDD proprioceptive neurons responsible for regulating dorsal head muscle contractions.

What physiological role do TRP-1 and TRP-2 play in the SMDD neurons? In general, TRP channels are nonselective Ca^2+^-permeable ion channels that permit Ca^2+^ influx in response to various stimuli [62]. In our Ca^2+^ imaging results, the SMDD neurons of *trp-1 trp-2* double mutants still show oscillating calcium dynamics, but rather than being synchronized with dorsal head/neck bending, they are synchronized with ventral head/neck bending. This means that the SMDD and SMDV neurons fire synchronously upon ventral head/neck bending. Thus, in contrast to TPR-4, which is required for the increased Ca^2+^ signals that appear in DVA neurons upon body bending, TRP-1 and TRP-2 are not required for Ca^2+^ signals in the SMDD neurons. Since the oscillating Ca^2+^ transients in the SMD neurons are completely abolished in the paralyzed *unc-54* mutants, SMD Ca^2+^ signals obviously originate from body movements. These Ca^2+^ influxes may arise from a yet unidentified proprioceptor in the SMD processes that run along the body. This proprioceptor would detect body bending/body muscle stretch and generate Ca^2+^ transients. In *Drosophila*, the TRP channels Nanchung, Inactive, and NompC are coexpressed in the auditory neurons of both the chordotonal and Johnston’s organs to detect and transduce sound vibrations [63–65]. Although these channels are all expressed in the auditory neurons and all function in hearing, the activities of NompC and the Nanchung–Inactive complex are independent of one another [63]. It is also possible that putative proprioceptors among the ventral nerve cord motor neurons detect body bending and cause waves of body muscle contractions [66, 67]. The VB1 ventral motor neurons are electrically coupled to the SMD neurons, and thus Ca^2+^ influx may propagate from these ventral motor neurons to the SMD neurons via gap junctions [26]. Recently, Fouad and colleagues showed that during forward locomotion, a midbody rhythmic signal or wave propagates from the midbody to the head via nonproprioceptive coupling [68]. Thus, we speculate that during forward locomotion, a rhythmic signal is transmitted from the VB1 motor neuron to the SMDV neurons. Moreover, the SMDV and SMDD neurons are also electrically coupled. Our working model is that during the forward movement, the oscillating calcium wave generated by midbody rhythm–generating units reaches at VB1 and causes ventral neck muscle contraction, and this calcium wave then transmits to the SMDV and SMDD, which synchronize the states of SMDV and SMDD together with that of VB1, and elicits ventral head/neck muscle contractions. Then, SMDD detects dorsal head/neck bending via TRP-1/TRP-2 and changes phase of SMDD calcium transients from those of SMDV, which initiates dorsal head muscle contractions via cholinergic transmission from SMDV to dorsal head muscles. TRP-1 and TRP-2 in SMDD neurons thus seem to play a role in detecting head/neck bending, and the SMDD neurons themselves seem to integrate information about head/neck bending and body bending. They separate the SMDD neurons from the circuit of the SMDV neurons and ventral muscle contractions, orchestrating dorsal head muscle contractions so the animal can steer straight (Fig 4H). In mice, ablation of proprioceptive systems also causes synchronized activation of muscles in the hip, knee, and ankles [69]. Together, we propose that synchronization of the locomotion circuits is a general consequence of the loss of function of proprioceptive neurons and receptor molecules, and distinct locomotion patterns require dynamic, homeostatic modulation of feedback signals between proprioceptive neurons and muscles.

## Materials and methods

### Strains

All strains were maintained at 20°C [29]. The N2 Bristol strain was used as a wild-type strain. To generate the *trp-1 trp-2* double mutants, *trp-1* (*sy690*) males were mated with *trp-2* (*sy691*) hermaphrodites, and the genomic deletion was confirmed by PCR (*trp-1*-deletion_1_F: GGCTAAGTTCCTGTCTACCAC, *trp-1*-deletion_2_F: TCTGCTACTCGTAGGGGCTT, *trp-1*-deletion_R: CTGTTGACAATGAGGATGAGAG; *trp-2*-deletion_1_F: CTACGCACTGATGACGTGGA. *trp-2*-deletion_2_F: AGTCACTGCTCAGAGCTACC, *trp-2*-deletion_R: AGTACGCAAACAACGACTACAG). All the mutants and transgenic strains used in this study are listed in Table 1.

**Table 1:** Mutants and transgenic strains used in this study.

Gene	Genotype	Strain
*acd-1*	*tm981*	-
*acd-2*	*ok1237*	RB1192
*acd-3*	*ok1335*	VC1047
*acd-4*	*ok1508*	RB1351
*acd-5*	*ok2657*	RB2005
*asic-1*	*ok415*	RB680
*asic-2*	*ok289*	RB557
*ced-11*	*tm1220*	-
Ex[*ceh-36*p-Δ1::GCaMP3.0 *+ unc-122*p::*dsRed*]	-	PY7548
Ex[*ceh-36*p-Δ1::GCaMP3.0; *unc-122*p::*dsRed + ceh-36*p-*Δ*1::*trp-1* cDNA; *unc-122*p::*gfp*]	*lskEx1013*	KHK1046
Ex[*ceh-36*p-Δ1::GCaMP3.0; *unc-122*p::*dsRed + ceh-36*p-*Δ*1::*trp-2* cDNA; *unc-122*p::*gfp*]	*lskEx1014*	KHK1032
*ctbp-1*	*ok498*	HRN017
*cup-5*	*ar465*	GS2477
*deg-1*	*u38*	TU38
*degt-1*	*ok3307*	VC2633
*del-1*	*ok150*	NC279
*del-2*	*tm6855*	-
*del-3*	*ok2613*	RB1979
*del-4*	*ok1014*	RB1064
*del-7*	*ok1187*	RB1156
*del-8*	*ok1357*	VC831
*del-9*	*ok2353*	RB1818
*del-10*	*ok1705*	RB1469
*delm-1*	*ok1226*	RB1177
*delm-2*	*ok1822*	RB1523
*egas-1*	*ok3497*	RB2521
*egas-2*	*ok1477*	VC975
*egas-3*	*ok1522*	RB1356
*egas-4*	*tm4826*	-
*egl-8*	*sa47*	JT47
Ex[*flp-7*p::*mCherry + unc-122*p::*gfp*]	*lskEx50*	KHK50
	*lskEx154*	KHK159
Ex[*flp-12*p::*gfp; flp-22*p*-Δ*4::*mCherry*]	*lskEx638*	KHK1085
Ex[*flp-22*p*-Δ*1::*gfp + unc-122*p::*dsRed*]	*lskEx515*	KHK745
	*lskEx516*	KHK746
Ex[*flp-22*p*-Δ*2::*gfp + unc-122*p::*dsRed*]	*lksEx514*	KHK744
	*lskEx526*	KHK757
Ex[*flp-22*p*-Δ*3::*gfp + unc-122*p::*dsRed*]	*lskEx544*	KHK782
Ex[*flp-22*p*-Δ*4::*gfp + unc-122*p::*dsRed*]	*lskEx546*	KHK784
	*lksEx550*	KHK789
Ex[*flp-22*p*-Δ*5::*gfp + unc-122*p::*dsRed*]	*lskEx517*	KHK747
	*lskEx518*	KHK748
Ex[*flp-22*p*-Δ*4::GCaMP3.0 *+ unc-122*p::*dsRed*]	*lskEx576*	KHK822
*lskIs6*[*flp-12*p::*gfp*];Ex[*flp-22*p*-Δ*4::*mCherry*]	*lskIs6*	KHK1259
Ex[*glr-1*p-*Δ*1::*mCherry*]	*lskEx547*	KHK785
	*lskEx585*	KHK835
*gon-2*	*q362*	EJ26
*gtl-1*	*ok375*	VC244
*gtl-2*	*n2618*	CZ9957
Ex[*gpa-4*p::GCaMP]	*lskEx1658*	KHK1658
Ex[*gpa-4*p::GCaMP]; Ex[*gpa-4*p::*trp-1* cDNA + *unc-122*p::*gfp*]	*lskEx1161*	KHK1659
Ex[*lad-2*p::*gfp + unc-122*p::*dsRed*]	*lskEx448*	KHK673
	*lskEx679*	KHK454
	*lskEx680*	KHK455
Ex[*lad-2*p*-Δ*1::*gfp + unc-122*p::*dsRed*]	*lskEx465*	KHK690
	*lskEx450*	KHK674
	*lskEx449*	KHK675
Ex[*lad-2*p*-Δ*1::ReaChR::mKate2 *+ unc-122*p::*dsRed*]	*lskEx1021*	KHK1472
Ex[*lad-2*p*-Δ*1::GCaMP; *myo-3*p:: ReaChR::mKate2 + *unc-122*p::*gfp*]	*lskEx1171*	KHK1671
*mec-4*	*u253*	TU253
*mec-10*	*e1515*	CB1515
*goeIs3*[*myo-3*p::GCaMP3.35]	*goeIs3*	HBR4
*goeIs3*[*myo-3*p::GCaMP3.35]; *lskEx1021*[*lad-2*p*-Δ*1::ReaChR::mKate2 *+ unc-122*p::*dsRed*]	*lskEx1021*	KHK1656
*ocr-1; ocr-2 osm-9*	*ak46;ak47 ky10*	FG125
*ocr-2*	*ak47*	CX4544
*ocr-3*	*ok1559*	RB1374
*ocr-4*	*vs137*	LX950
*plc-1*	*rx1*	PS4112
*plc-2*	*ok761*	PS4886
*plc-3*	*tm1340*	PS4886
*plc-4*	*ok1215*	RB1173
*pkd-2*	*sy606*	PT8
*trp-1*	*sy690*	TQ225
*trp-1*p::*gfp*	*kyIs123*	OH1358
*Is*[*trp-1*p::*gfp*]; Ex[*flp-22*p::*mCherry + unc-122*p::*gfp*]	*lskEx383*	KHK397
*Is*[*trp-1*p::*gfp*]; Ex[*trp-2*p::*mCherry*]	*lskEx441*	KHK656
*trp-1;* Ex[*flp-22*p*-Δ*4::GCaMP3.0 *+ unc-122*p::*dsRed*]	*lskEx576*	KHK1026
*trp-2*	*sy691*	TQ194
Ex[*trp-2*p::*mCherry; flp-22*p::*gfp + unc-122*p::*gfp*]	*lskEx383*	KHK667
*trp-2;* Ex[*flp-22*p*-Δ*4::GCaMP3.0 *+ unc-122*p::*dsRed*]	*lskEx576*	KHK1027
*trp-1 trp-2*	*sy690 sy691*	KHK641
*trp-1 trp-2;* Ex[*trp-1*p::*trp-1* cDNA *+ unc-122*p::*dsRed*]	*lskEx396*	KHK410
*trp-1 trp-2;* Ex[*trp-1*p::*trp-2* cDNA *+ unc-122*p::*dsRed*]	*lskEx528*	KHK760
*trp-1 trp-2;* Ex[*flp-22*p*-Δ*4::GCaMP3.0 *+ unc-122*p::*dsRed*]	*lskEx576*	KHK1060
*trp-1 trp-2;* Ex[*flp-22*p*-Δ*4::GCaMP3.0 *+ glr-1*p*-Δ*1::*trp-1* cDNA *+ unc-122*p::*dsRed + unc-122*p::*gfp*]	*lskEx627*	KHK887
*trp-1 trp-2;* Ex[*flp-22*p*-Δ*4::*trp-1* cDNA *+ unc-122*p::*dsRed*]	*lskEx565*	KHK810
	*lskEx566*	KHK811
*trp-1 trp-2;* Ex[*flp-22*p*-Δ*4::*trp-2* cDNA *+ unc-122*p::*dsRed*]	*lskEx622*	KHK883
	*lskEx623*	KHK884
*trp-1 trp-2;* Ex[*flp-22*p*-Δ*4::*trp-4* cDNA *+ unc-122*p::*dsRed*]	*lskEx615*	KHK876
	*lskEx616*	KHK877
	*lskEx617*	KHK878
*trp-1 trp-2;* Ex[*flp-22*p*-Δ*4::*trpγ* cDNA *+ unc-122*p::*dsRed*]	*lskEx762*	KHK1078
	*lskEx763*	KHK1079
	*lskEx764*	KHK1080
*trp-1 trp-2;* Ex[*flp-7*p::*trp-1* cDNA *+ unc-122*p::*dsRed*]	*lsk567*	KHK812
	*lsk568*	KHK813
*trp-1 trp-2;* Ex[*flp-7*p::*trp-2* cDNA *+ unc-122*p::*dsRed*]	*lskEx583*	KHK833
	*lskEx1159*	KHK1657
*trp-1 trp-2;* Ex[*glr-1*p*-Δ*1::*trp-1* cDNA *+ unc-122*p::*dsRed*]	*lskEx570*	KHK815
	*lskEx571*	KHK816
*trp-1 trp-2;* Ex[*glr-1*p*-Δ*1::*trp-2* cDNA *+ unc-122*p::*dsRed*]	*lskEx630*	KHK891
	*lskEx631*	KHK892
*trp-1 trp-2;* Ex[*myo-3*p::GCaMP3.35 *+ unc-122*p::*dsRed*]	*goeIs3*	KHK1110
*trp-4*	*sy695*	TQ296
*trp-4;* Ex[*twk-16*p::*trp-1* cDNA *+ unc-122*p::*dsRed*]	*lskEx632*	KHK893
	*lskEx633*	KHK894
*trp-4;* Ex[*twk-16*p::*trp-2* cDNA *+ unc-122*p::*dsRed*]	*lskEx614*	KHK875
*trp-4;* Ex[*twk-16*p::*trp-4* cDNA *+ unc-122*p::*dsRed*]	*lskEx605*	KHK864
	*lskEx606*	KHK865
*trpa-1*	*ok999*	RB1052
*trpa-2*	*tm3085*	M05B5.6
*trpl-1*	*ok2305*	RB1787
*trpl-2*	*ok2433*	RB1883
*trpl-3*	*tm4643*	-
*trpl-5*	*ok1507*	RB1350
*unc-13*	*e1091*	CB1091
*unc-13;* Ex[*flp-22*p*-Δ*4::GCaMP3.0 *+ unc-122*p::*dsRed*]	*lskEx576*	KHK842
*unc-31*	*e928*	CB928
*unc-31;* Ex[*flp-22*p*-Δ*4::GCaMP3.0 *+ unc-122*p::*dsRed*]	*lskEx576*	KHK863
*unc-54*	*e1092*	CB190
*unc-54;* Ex[*flp-22*p*-Δ*4::GCaMP3.0 *+ unc-122*p::*dsRed*]	*lskEx914*	KHK1306
*unc-89*	*e1460*	CB1460

### Molecular biology and transgenic worms

All the constructs created in this study were inserted into the pPD95.77 vector [70]. For the *flp-22* and *lad-2* promoter analysis, 2.7-kb and 7.3-kb promoter regions were amplified by PCR from N2 genomic DNA and used to generate *flp-22*p::*gfp* and *lad-2*p::*gfp* as previously described [38, 71]. Deletions in the *flp-22* and *lad-2* promoter reporter constructs were generated by using various enzymes or PCR fusions. To generate transgenic worms, 50 ng of each reporter construct was injected, with 50 ng of *unc-122*p::*dsRed* as an injection marker. Among the various *flp-22* promoter fragments, *flp-22*p*-Δ*4 was used as an SMD neuronal marker. A 2.6-kb fragment of the *trp-1* promoter and a 2.9-kb fragment of the *trp-2* promoter from the start codon were amplified by PCR and inserted into the pPD95.77 vector. For the rescue experiments, *glr-1*p-*Δ*1 (for SMDD; a gift from Hannah Nicholas) [39], *flp-7*p (for SMDV) [38], *flp-22*p*-Δ*4 (for SMDD/V), and *twk-16*p (for DVA; a gift from Shawn Xu) [13] were fused with a *trp-1* cDNA, a *trp-2* cDNA, a *trp-4* cDNA (gift from Shawn Xu) [36], and a *trpγ* cDNA (a gift from Craig Montell) [8]. The *trp-1* cDNA and the *trp-2* cDNA were amplified by PCR from a cDNA library. With 50 ng of *unc-122*p::*dsRed* as an injection marker, 0.5 ng of the *trp-1*, *trp-2*, *trp-4*, and *trpγ* cDNAs under the control of various promoters were injected into *trp-1 trp-2* double mutants or *trp-4* mutants. For ectopic expression of *trp-1* cDNA or *trp-2* cDNA in AWC, *ceh-36*p-*Δ*1 (a gift from Piali Sengupta) was fused with the *trp-1* cDNA or *trp-2* cDNA in the pPD95.77 vector, and *gpa-4*p was fused with the *trp-1* cDNA in the pPD95.77 vector for ectopic expression in AWA, and ASI. 0.5 ng of each transgene was injected with 50 ng of *unc-122*p::*dsRed* as an injection marker. *lad-2*p-*Δ*1 and *myo-3*p were inserted into the ReaChR::mKate2 vector (a gift from Henrik Bringmann) [42, 43], and 100 ng of each transgene was injected with 50 ng of *unc-122*p::*dsRed* or *unc-122*p::*gfp* as an injection marker, respectively.

### Behavior tracking and phenotype analysis

To record animal locomotion, NGM agar plates were coated with 600 μl of *Escherichia coli* OP50, incubated for 3 h, and allowed to fully dry. Bacterial lawn plates were prepared the day before behavior recordings. Well-fed young adult hermaphrodite worms were used to leave trajectories on bacterial lawn plates and recorded under a Leica High-performance Fluorescence Stereomicroscope (M205FA) using the Leica Application Suite Advanced Fluorescence Lite 3.5 software. To quantify the turning angle of the worm paths, 5 min of behavior video were collected into 30 s–interval images. A total of 10 images from each worm were used to measure the dorsal or ventral angles from the dorsoventral body positions during forward movement. From each image, at least 3 ventral and dorsal angles were measured using Image J, and the angle values were averaged. The averaged dorsal angles were then subtracted from the averaged ventral angles to obtain a turning angle. The turning angles were then color-coded along a brown-to-blue scale. Ventral circling was defined as dark brown, and dorsal circling was defined as dark blue. Twenty worms for each strain were analyzed to generate a locomotion pattern heat map. To measure wave length and wave width, the Leica Application Suite Advanced Fluorescence Lite 3.5 software was used to capture images of worm tracks on the bacterial lawn. Wave width was defined as the peak-to-trough distance of the sine wave, and wavelength was defined as the peak-to-peak distance of the sine wave. The average of 6 consecutive wave widths and wave lengths from each worm track was quantified. At least 50 worms for each strain were measured and analyzed.

### In vivo calcium imaging in freely moving condition

Well-fed young adult–stage worms were used to observe SMD and head muscle calcium activities. For freely moving conditions, each worm was transferred onto a 2% agarose pad on a glass slide and placed on a coverslip. Fluorescence time lapse images were acquired over the course of 80 s under a Zeiss Axio observer A1 with a 20× objective using the Image J software. At least 100 frames (20 s) of continuous forward movement from each worm were selected and analyzed with a customized program that automatically normalizes SMD fluorescence intensity and subtracts the background. Head muscle activities were obtained together with SMD neuronal activities and analyzed as described above. To quantify SMD neuronal activity in the *unc-54* mutants induced by head bending, a platinum wire was used to manually bend the heads of worms on the NGM agar plate. The resulting calcium intensities were observed under a Zeiss SteREO Discovery V8.

### Laser ablation

Young adult–stage *flp-22*p*-Δ*4::*mCherry; flp-12*p::*gfp* transgenic worms were anesthetized with 1 M sodium azide on the 2% agar pad. Transgenic worms coexpressing markers of both the SMD and SMB neurons (*flp-22*p*-Δ*4::*mCherry; flp-12*p::*gfp*) were used for precise ablation of the SMD cell bodies because the SMD and SMB neurons are so close to one another. After ablation, animals that exhibited GFP expression in the SMB neurons were selected [27, 37]. A commercial femtosecond (approximately 120 fs) pulse laser system (Insight Deepsee Dual, Spectra physics), employed as a light source (790 nm) for nonlinear imaging in an Olympus IX83 microscope platform, was also used to kill the SMDD or SMDV neurons (56 mW illumination during 35 s). After these ablations, the worms were transferred to NGM agar plates immediately and allowed to recover for 14 h at 15°C. Mock-ablated worms were placed on the same agar pad to expose them to light and allowed to recover on separate NGM agar plates. Locomotion was observed and recorded over 5 min with the Leica High-performance Fluorescence Stereomicroscope M205FA using the Leica Application Suite Advanced Fluorescence Lite 3.5 software. After the behavioral recordings, the expression of *flp-22*p*-Δ*4::*mCherry* in the SMD neurons and *flp-12*p::*gfp* in the SMB neurons were confirmed under a Zeiss Axio observer A1 with a 40× objective. Only worms that specifically lost fluorescence of *flp-22*p*-Δ*4::*mCherry* in their SMD neurons but exhibited expression of *flp-12*p::*gfp* in the SMB neurons were counted as ablated worms.

### Coherent anti-Stokes Raman scattering microscopy

Young adult stage of wild-type and *trp-1 trp-2* double-mutant animals were anesthetized with 100mM sodium azide on the 2% agar pad and placed on a top of an agarose pad. The label-free images were obtained under the coherent anti-Stokes Raman scattering microscopy, followed by the setting of the microscopy as previously described [50].

### Optogenetic experiments in freely moving condition

L4 transgenic worm larvae expressing ReaChR::mKate2 transgenes under the control of indicated promoters were transferred 12 h before the assay to either normal OP50 plates or OP50-retinal plates containing 1 mM all-trans-retinal (ATR, Sigma). OP50-retinal plates were prepared by seeding 200 μl OP50 with 2 μl 100 mM ATR (Sigma) in 100% ethanol. To stimulate ReaChR, we illuminated the NGM plates with 565 nm LED at roughly 0.05 mW/mm as measured with an optical power/energy meter. We recorded at least 40 young adult hermaphrodites per strain in the presence or absence of ATR under a custom automated worm-tracking system. The animals were allowed to move on the NGM agar plates for at least 1 min. Then, the recordings began with 10 s in the absence of green light, followed by 10 s of green light stimulation, and finally another 10 s without green light. Circling behavior was determined within 1 s after supplying the green light stimulus.

### Cross-correlation analysis

The JMP10 software (SAS) was used as described to analyze time series of head bending angles and calcium fluorescence intensities for a cross-correlation analysis [40]. The time lag was 20 s, and head position was used as an input. Peak correlations are the correlation values between head bending and SMD activity at lag 0 s and are compared using the ANOVA test.

## Supporting information

S1 Fig Phenotypic mutant screen of TRP and DEG/ENaC channel family genes analyzing wave width. *n* = 50 for each. Numerical values that underlie the graph are shown in S1 Data. Error bars indicate SEM. *** indicates significant differences from wild type at *p* < 0.001 (one-way ANOVA test followed by the Tukey post hoc test). DEG/ENaC, degenerin/epithelial sodium channels; TRP, transient receptor potential.
(TIF)

S2 Fig Phenotypic mutant screen of TRP and DEG/ENaC channel family genes analyzing wave length. *n* = 50 for each. Numerical values that underlie the graph are shown in S1 Data. Error bars indicate SEM. ** and *** indicate significant differences from wild type at *p* < 0.01 and *p* < 0.001, respectively (one-way ANOVA test followed by the Tukey post hoc test). DEG/ENaC, degenerin/epithelial sodium channels; TRP, transient receptor potential.
(TIF)

S3 Fig Phenotypic mutant screen of TRP and DEG/ENaC channel family genes analyzing turning angle. Heat maps and the average turning angle of TRP and DEG/ENaC channel gene-defective mutants. *n* = 20 for each. TRP channel mutants are indicated in orange, and DEG/ENaC channel mutants are indicated in yellow. Numerical values that underlie the graph are shown in S1 Data. Error bars indicate SEM. * and ** indicate significant differences from wild type at *p* < 0.05 and *p* < 0.01, respectively (one-way ANOVA test followed by the Tukey post hoc test). DEG/ENaC, degenerin/epithelial sodium channels; TRP, transient receptor potential.
(TIF)

S4 Fig Phenotypic analysis of *trp-1*, *trp-2*, and *trp-1 trp-2* double-mutant animals. Average wave length and wave width for the indicated genotypes: wild type, *trp-1* (*sy690*), *trp-2* (*sy691*), *trp-1 trp-2* (*sy690 sy691*). *n* = 50 for each. Numerical values that underlie the graph are shown in S1 Data. Error bars indicate SEM. *** indicates significant differences from wild type at *p* < 0.001 (one-way ANOVA test followed by the Tukey post hoc test).
(TIF)

S5 Fig Turning angle of *trp-1 trp-2* mutants depending on food condition. Heat maps and the average turning angle of *trp-1 trp-2* mutants in the presence or absence of food. *n* = 20 for each. Numerical values that underlie the graph are shown in S1 Data. Error bars indicate SEM. *** indicates significant differences from wild type at *p* < 0.001 (one-way ANOVA test followed by the Tukey post hoc test).
(TIF)

S6 Fig Turning angle of *trp-1 trp-2* mutants during reversals. At least 3 turning angles during reversals were measured for individual worms. *n* = 20 for each. Numerical values that underlie the graph are shown in S1 Data. Error bars indicate SEM.
(TIF)

S7 Fig Promoter analysis of the *flp-22* and *lad-2* genes. **(A)** The percentage of transgenic animals expressing each *gfp* reporter construct in the SMDD and SMDV neurons. The strength of the GFP expression is indicated by the number of + symbols. The regions deleted from each promoter are indicated by a solid line. At least 2 independent extrachromosomal lines for each construct were examined. *n* = 50 for each. **(B)** Representative image showing the expression pattern for a *flp-22*p*-Δ*4::*mCherry* transgenic animal. The SMD, URX/CEP, and ASG cell bodies are indicated. Scare bar: 25 μm. Anterior is to the left. **(C)** The percentage of transgenic animals expressing 2 *gfp* reporter constructs in several neurons including SMD, SAA, ALN, PLN, and SDQ. The strength of the GFP expression is indicated by the number of + symbols. The regions deleted from each promoter are indicated by solid lines. At least 2 independent extrachromosomal lines for each construct were examined. *n* = 50 for each. GFP, green fluorescent protein.
(TIF)

S8 Fig Analysis of transgenic strains that drive gene expression under the control of *glr-1*p-Δ1 and *flp-7* promoters. (A) Heat maps for the indicated genotypes. *n* = 20 for each. (B) Expression pattern analysis of *flp-7*p::*mCherry* and *glr-1*p-*Δ*1::*mCherry* in the SMD neurons. Blue, brown, and yellow color indicate *mCherry* expression in SMDD, SMDV, and SMDD/V, respectively. *n* = 50 for each. Numerical values that underlie the graph are shown in S1 Data. Error bars indicate SEM.
(TIF)

S9 Fig Traces of calcium transients in the SMD neurons and the corresponding head bending in wild type and *trp-1 trp-2* double-mutant animals. Gray bar represents the duration of the dorsal head bending. *n* = 10 for each.
(TIF)

S10 Fig Analysis of SMD calcium activity during head bending. (A) GCaMP fluorescence ratio between SMDV and SMDD. *n* = 10 for each. Average of SMDV GCaMP fluorescence intensity was divided by average of SMDD GCaMP fluorescence intensity. Numerical values that underlie the graph are shown in S1 Data. Error bars indicate SEM. ** indicates significant differences from wild type at *p* < 0.01 (one-way ANOVA test followed by the Tukey post hoc test). (B) Cross-correlations and peak correlations between SMD Ca^2+^ dynamics and head bending of *trp-1 (sy690)* and *trp-2 (sy691)* single mutants. Blue solid and red dashed lines indicate SMDD and SMDV neuronal activity with head bending, respectively. Peak correlation is the correlation value between head bending and SMD activity at lag 0 s. *n* = 10 for each. Numerical values that underlie the graph are shown in S1 Data. Error bars indicate SEM.
(TIF)

S11 Fig Optogenetical activation of the SMD neurons induce muscle activation. Representative images showing Ex[*lad-2*p-*Δ*1::ReaChR; *myo-3*p::GCaMP] transgenic animals upon green light stimulation in the presence and absence of retinal (ATR; top) and the GCaMP3 fluorescence intensity of head muscles (bottom). Black rectangles indicate the region at which GCaMP3 intensity was measured. *n* = 23. Numerical values that underlie the graph are shown in S1 Data. Error bars indicate SEM. *** indicates significant differences from wild type at *p* < 0.001 (one-way ANOVA test followed by the Tukey post hoc test). ATR, all-trans-retinal.
(TIF)

S12 Fig Traces of calcium transients in the SMD neurons and the corresponding head muscle activity in wild-type and *trp-1 trp-2* double-mutant animals. Gray bar represents the duration of the dorsal head bending. *n* = 6 for each.
(TIF)

S13 Fig Body wall muscle morphology of *trp-1 trp-2* double mutants. Label-free images of body wall musculature in wild-type, *trp-1 trp-2* double-mutant, and *unc-89* mutant animals. The images were obtained with coherent anti-Stokes Raman scattering microscopy assisted by sum-frequency generation. Left images are the merged image of each genotype, and white rectangles represent the regions that were enlarged on next column. Scale bars are indicated in each image that was magnified.
(TIF)

S14 Fig Cross-correlations and peak correlations of the ASI and AWA neurons between neuronal calcium responses and nose bending. Peak correlation value is obtained from lag 0 of cross-correlation. *n* = 6 for each. Numerical values that underlie the graph are shown in S1 Data. Error bars indicate SEM. ** indicates significant differences from control at *p* < 0.01 (one-way ANOVA test followed by the Tukey post hoc test).
(TIF)

S1 Video Sinusoidal locomotion of a wild-type animal on an NGM agar plate. The animal moves forward toward the right. NGM, nematode growth medium.
(AVI)

S2 Video Ventral circling locomotion of a *trp-1 trp-2* double mutant on an NGM agar plate. The animal moves in circles toward the ventral side of the body on a bacterial lawn. NGM, nematode growth medium.
(AVI)

S3 Video SMD Ca2+ dynamics of a wild-type animal expressing Ex[*flp-22*p-Δ4::GCaMP3.0] during head bending. The dorsal side of the body is up, and the ventral side of the body is down. The SMDD and SMDV cell bodies are indicated at the beginning of the movie with white arrowheads.
(AVI)

S4 Video SMD Ca2+ dynamics of a *trp-1 trp-2* double mutant animal expressing Ex[*flp-22*p-Δ4::GCaMP3.0] during head bending. The dorsal side of the body is up, and the ventral side of the body is down. The SMDD and SMDV cell bodies are indicated at the beginning of the movie with white arrowheads.
(AVI)

S5 Video Light stimulation of an Ex[*lad-2*p-Δ1::ReaChR::mKate2] transgenic animal to activate SMD. The green light exposure lasts 10 s. The dorsal side of the body is up, and the ventral side of the body is down. The animal moves forward toward the right.
(AVI)

S1 Data (XLSX)

## Abbreviations

ATR: all-trans-retinal
bd: bipolar dendrite
CTBP-1: C-terminal binding protein 1
da: dendritic arborization
DEG/ENaC: degenerin/epithelial sodium channels
TRP: transient receptor potential
TRPA: transient receptor potential Ankyrin
TRPC: transient receptor potential cation
TRPM: transient receptor potential mucolipin
TRPN/NompC: transient receptor potential no mechanical potential C
TRPV: transient receptor potential vanilloid
VNC: ventral nerve cord
