# Supplementary figures and images for: A sensory-motor neuron type mediates proprioceptive coordination of steering in C. elegans via two TRPC channels
Source: PLoS Biol. 2018 Jun 8;16(6):e2004929. doi: 10.1371/journal.pbio.2004929 (PMC6010301; doi:10.1371/journal.pbio.2004929)

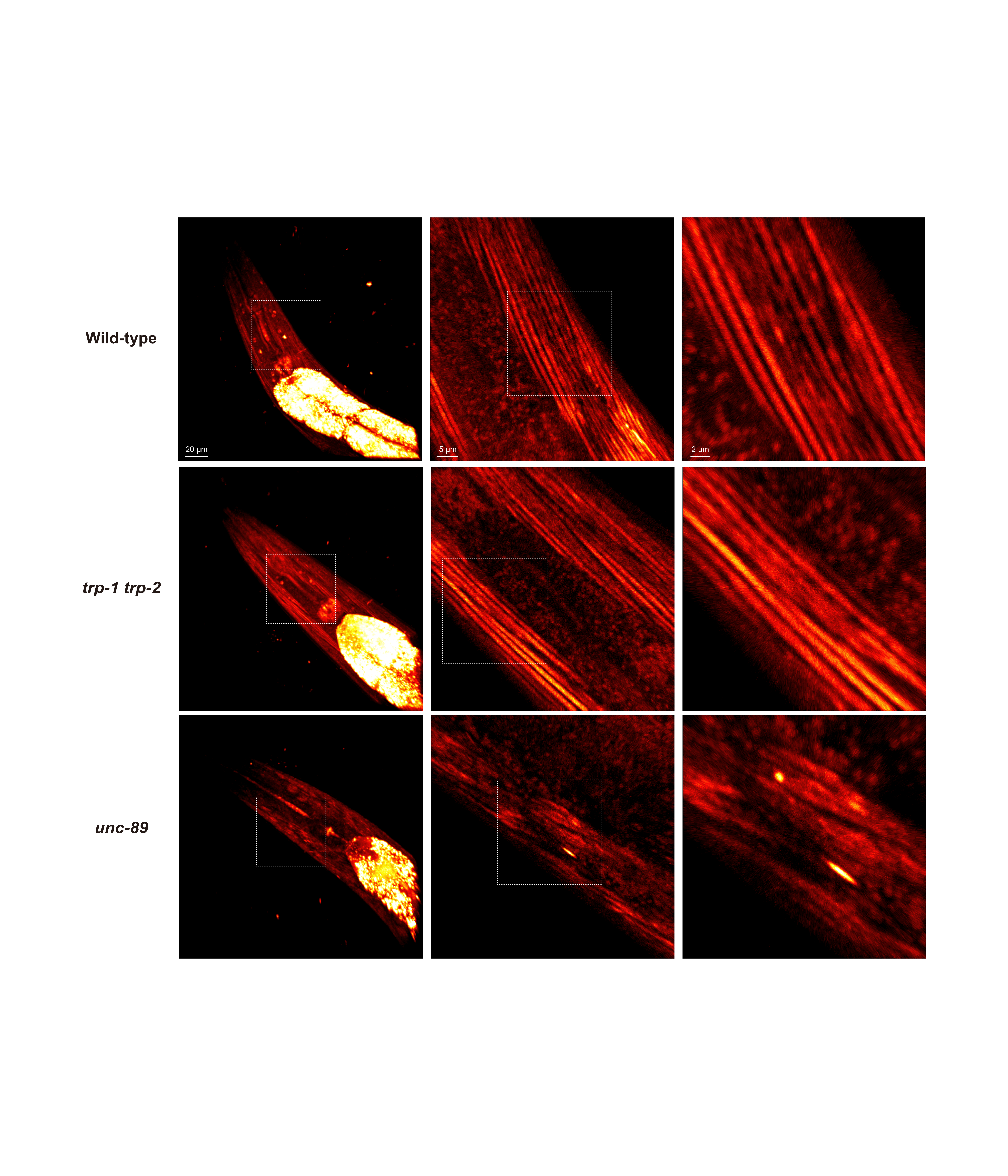

Supplement: S13 Fig — Label-free images of body wall musculature in wild-type, trp-1 trp-2 double-mutant, and unc-89 mutant animals. The images were obtained with coherent anti-Stokes Raman scattering microscopy assisted by sum-frequency generation. Left images are the merged image of each genotype, and white rectangles represent the regions that were enlarged on next column. Scale bars are indicated in each image that was magnified. (TIF) [file pbio.2004929.s013.tif]
